# Food addiction as a new piece of the obesity framework

**DOI:** 10.1186/s12937-016-0124-6

**Published:** 2016-01-13

**Authors:** Jose Manuel Lerma-Cabrera, Francisca Carvajal, Patricia Lopez-Legarrea

**Affiliations:** 1 Centro de Investigacion Biomedica, Facultad de Ciencias de la Salud, Universidad Autonoma de Chile, Santiago, Chile; 2 Depto de Psicología y Sociología. Facultad de Ciencias Sociales y Humanas, Universidad de Zaragoza, Teruel, España

**Keywords:** Obesity, food addiction, neuropeptides, palatable food, binge eating

## Introduction. Obesity today

Obesity has become a major public health burden worldwide due to the huge social and economic impact derived from its related comorbidities [1]. Excessive body weight has been estimated to account for 16 % of the global burden disease [2] and according to World Health Organization estimates, over 600 million adults are obese worldwide Obesity is described as a multi-etiological disorder and several factors have been shown to be involved in its onset and development [1]. Despite the important progression in the study of obesity, prevalence rates continue to increase, suggesting that additional elements must be involved in the pathogenesis of this disease. Moreover, even if weight loss programs are effective, keeping the weight off continues to be an almost insurmountable challenge [3]. In this context, new theories are arising regarding food intake. Understanding obesity as a food addiction is a novel approach that has garnered considerable attention. Some studies have shown an association between mood and the overall dietary pattern including specific nutrients [4]. Recent research also shows that palatable and high calorie food may have addictive potential. Subjects chronically eat some foods in amounts larger than needed for staying healthy, which shows a loss of control in food behaviour [5]. Additionally, a 40 % prevalence of food addiction has been shown in obese individuals seeking bariatric surgery [6]. All these traces indicate that there may be a potential relationship between behaviour and weight gain.

## New theories about obesity: obesity as food addiction

In recent years, there has been an increase in scientific evidence showing both neurobiological and behavioral relationships between drugs and food intake. Basic research using animal and humans models has shown that certain foods, mainly highly palatable foods, have addictive properties. In addition, exposure to food and drugs of abuse have shown similar responses in the dopaminergic and opioid systems. These similarities between food and drugs have given rise to the hypothesis of food addiction.

### Food intake and brain reward circuits

The dopaminergic system is involved in a large number of behaviors including reward processing and motivated behavior. Thus, all drugs of abuse increase the extracellular concentration of dopamine (DA) in the striatum and associated mesolimbic regions [7]. Di Chiara’s group has extensively showed that addictive drugs (e.g. amphetamine and cocaine) increase extracellular DA in the nucleus accumbens (NAc), a primary site for reinforced behaviors [7]. Likewise, microdialysis has shown that exposure to rewarding food stimulates dopaminergic transmission in the NAc [8].

Furthermore, neuroimaging studies show that our brain response is similar in the presence of food and drug abuse: increased cell activation in the NAc, the brain’s pleasure center [9–11]. Neuroimaging studies in humans have also shown similarities between obesity and addiction. For example, both obesity and addiction are associated with fewer D2 dopamine receptors in the brain [12, 13], suggesting that they are less sensitive to reward stimuli and more vulnerable to food or drug intake. Thus, for example, individuals with the largest body mass index (BMI) had the lowest D2 values [13].

Specifically, this reduction in striatal D2 density correlates with reduced metabolism in cerebral areas (prefrontal and orbitofrontal cortex) that exert inhibitory control over consumption [12]. Thus, obese subjects show greater activation of reward and attention regions than normal weight subjects do in response to palatable food images versus control images [14, 15]. This observation suggests that a deficit in reward processing is an important risk factor for the impulsive and compulsive behaviors showed by obese individuals. Taken together, these data could explain why in obesity and drug addiction the consummatory behaviors persist despite negative social, health and financial consequences. All these neurobiological data suggest that obesity and drug addiction may share similar neuroadaptive responses in brain reward circuits or action mechanisms.

### The role of nutrition neuropeptides in addiction

The idea that neuropeptides involved in metabolic regulation are also involved in modulating the neurobiological responses to drugs of abuse has received a great deal of attention in recent literature [16, 17]. Several studies have shown that exposure to drugs of abuse significantly alters the functionality of numerous neuropeptides systems. On the other hand, compounds that target these neuropeptides systems play an important role in modulating the neurobiological responses to drugs of abuse. For example, the melanocortins (MC) and orexins system, which plays an important role in food intake, is also involved in drug use. Moreover, the brain expression of these neuropeptides is altered after drug binge-like consumption [18–20] or palatable substances (caloric and non-caloric) [21]. Central administration of Agouti-related peptide, an MC antagonist, activates midbrain dopamine neurons and induces consumption of fat enriched foods [22]. Taken together, this data could explain why some kinds of food are so often overconsumed.

Regulatory mechanisms for food intake can be homeostatic –biological need– but also hedonic [23]. This idea is supported by the fact that people continue eating even when energy requirements have been met. However, it is noteworthy that these systems (hedonic versus homeostatic) are not mutually exclusive, but will have multiple interconnections [24]. Homeostatic regulators of hunger and satiety, such as ghrelin, leptin and insulin, could mediate between the homeostatic and hedonic mechanisms of food intake influencing the dopaminergic system [25, 26]. Leptin is perhaps the most widely studied biological factor in relation to food intake control. Although it is secreted by the adipose tissue, leptin receptors are expressed on midbrain dopamine neurons [27]. Leptin infusion into the tegmental ventral area, a reward system brain area, decreases food intake and inhibits the activity of dopamine neurons [28]. Thus, current evidence suggests that mesolimbic dopamine pathways could mediate the effect of leptin on food intake.

Therefore, theories of “food addiction” indicate that certain highly processed foods can have a high addictive potential and may be responsible for some cases of obesity and eating disorders [29, 30]. Recently, it have been shown that subjects showing a compulsive overeating consume higher amounts of some macronutrients (fats and proteins) compared with non-food-addicted subjects [31, 32]. It is well established that hyperphagia induced by consumption of fat enriched food and refined sugars is influenced by mesolimbic and nigrostriatal dopaminergic inputs. For example, consumption of highly palatable food, especially sugar, entails the release of endogenous opioids in the NAc [33, 34] and activates the dopaminergic reward system [35]. In addition, the rats exposed to intermittent access to sugar solution show some components of addiction such as escalation of daily sugar intakes, withdrawal signs, craving and cross-sensitization to amphetamine and alcohol [36]. These data suggest that certain foods are potentially rewarding and can trigger addictive-like behaviors in laboratory animals and humans.

## How to evaluate food addiction

As mentioned before, obesity is a heterogeneous disease influenced by multiple factors. This review has shown how an addictive process may play a role in binge eating and obesity. Thus, food addiction could be a factor contributing to overeating and then to obesity. However, for the scientific community the concept of food addiction is still a controversial topic [5, 37, 38]. One of the arguments to question the validity of food addiction hypothesis is that although neurobiological studies have identified shared brain mechanisms of food and drugs, there are substantial differences too [37]. Also, the pattern of brain activation of obese individuals and binge-eaters compared with controls is inconsistent [38]. Finally, other critical remarks argue that most of the studies that support the existence of food addiction are restricted to animal models [5]. Bearing in mind this criticism, future research is required to more extensively study the validity of food addiction in humans. Therefore, to evaluate this hypothesis of “food addiction” and its contribution to eating disorders it becomes necessary to have valid and reliable instruments to operationalize food addictive behaviors.

A tool to identify individuals showing symptoms of “dependence” to certain foods has been recently developed. Gearhardt and cols. elaborated in 2009 the Yale Food Addiction Scale (YFAS) [39]. This scale has been used in most of the research related to the concept of food addiction and has been translated into several languages, such as, French, German, Italian, Spanish or Dutch. The instrument is a 25-item questionnaire grouped under criteria that resemble the symptoms of substance dependence as outlined in the Diagnostic and Statistical Manual of Mental Disorders IV. The scale includes items that assess specific criteria, such as loss of control over consumption, a persistent desire or repeated unsuccessful attempts to quit, continued use despite physical and psychological problems, and clinically significant impairment or distress, among others. The most common symptoms of food addiction are loss of control over consumption, continued use despite negative consequences, and inability to cut down despite the desire to do so [40].

Studies using the YFAS have found that patients scoring high in the scale show more frequently binge eating episodes [22, 41, 42]. In turn, prevalence of food addiction diagnosed using YFAS was 5.4 % in general population [31]. However, food addiction increased with obesity status range between 40 % and 70 % in individuals with binge eating disorder [42], compulsive-overeating [43] or bulimia nervosa [6]. Furthermore, individuals with high food addiction scores were found to have comparable responses when viewing food images as individuals with drug dependence viewing drug cues. They showed elevated activation in reward circuitry (anterior cingulate cortex, dorsolateral prefrontal cortex and amygdala) in response to food cues and reduced activation in inhibitory regions (medial orbitofrontal cortex) in response to food intake [29].

Interestingly, the prevalence of food addiction was positively related to measures of adiposity (e.g. body fat, BMI) [31, 44]. These data suggest that food addiction is likely an important factor in the development of human obesity and that it is associated with the severity of obesity from normal to obese individuals. In fact, obese people showing a worse weight loss response to treatment [41] and greater weight gain after undergoing bariatric surgery [45] obtain higher YFAS scores. Thus, weight-loss treatments should consider the role of food addiction as a psychological factor underlying difficult weight management situations.

On the other hand, some personality traits, such as impulsivity, have been associated with alcohol and drug misuse [46]. In the context of food addiction, recent research has demonstrated that obese individuals scoring high in YFAS were more impulsive and displayed greater emotional reactivity than obese controls [22]. These findings suggest that a food addiction construct shows a psycho-behavioral profile similar to conventional drug abuse disorders.

However, although food addiction constructs exists, it is highly unlikely that all foods have addictive potential. Manufacturing industries have designed processed foods by adding sugar, salt, or fat, which can maximize the reinforcing properties of traditional foods (fruits, vegetables). The high palatability (hedonic value) that this kind of processed food offers, prompts subjects to eat more. Thus, certain processed food may have a high addictive potential and be responsible for some eating disorders such as obesity [30, 40]. Although there is little evidence in humans, animal models suggest that processed food is associated with addictive-like eating. For example, Avena and cols. showed that excessive intake of sugar causes neurochemical (increased release of dopamine and acetylcholine in NAc) and behavioral (increased intake of sugar after a period of abstinence and cross-sensitivity to drugs of abuse) signs of dependence [47]. These findings suggest that overconsumption of highly processed food, but not standard rat chow, produce some addictive-like characteristics. Also, it has been shown that overconsumption of palatable food triggers down-regulation of striatal D2 receptors expression in the same way that drugs do [48], which suggests that obesity and drug addiction may share an underlying hedonic mechanism, as noted above.

Nevertheless, not everyone exposed to palatable food environments develops obesity. Knowing the biological and/or behavioral motives or reasons why people eat highly palatable foods could help explain the susceptibility or resilience with respect to obesity. Thus, by identifying why people begin to eat these kinds of food it could be possible to design appropriate “personalized” treatments to combat obesity. The Palatable Motives Eating Scale (PEMS) is a validated and robust scale to identify motivations for eating highly-palatable foods [49]. The scale allows detecting motives for eating tasty food: social (e.g., to celebrate a special occasion with friends), coping (e.g., to forget about your problems), reward enhancement (e.g., because it gives you a pleasant feeling) and conformity (e.g., because your friends or family want you to eat or drink these foods or drinks). Moreover, PEMS have a good convergent validity with YFAS scores. It makes it possible to evaluate different food addiction constructs. While the YFAS probes the consequences of consuming highly palatable foods, the PEMS probes the motives for such consumption.

Two examples of scales (YFAS and PEMS) to evaluate food addiction have been shown.

## Conclusion

As indicated above, obesity has become a major public health problem worldwide. Therefore, finding efficient strategies to fight this disease represents a big challenge for the international scientific community. Studying the possible role of food addiction in humans as an influencing factor in excessive food intake is attracting attention. More so, considering the interesting results obtained with animals. It is known that some cases of excessive food intake do not respond to physiological needs but to a psychological behavioural component that needs to be identified. Finding this component would allow the inclusion of behavioural therapy among the cornerstones of obesity treatment, thus achieving a multidisciplinary approach in accordance to the multifactorial origin of the obesity. This more realistic grasp may allow applying effective treatments, leading not only to greater weight loses, but also to a better chance of keeping the lost weight off. YFAS and PEMS tools offer a rigorous way to evaluate whether an addictive process contributes to certain eating disorders, such as obesity and binge eating. However, further research is needed in order to evaluate the food addiction hypothesis and its relationship to eating disorders. It is necessary to study the effect of psychological, behavioral, cognitive and physiological factors in the food addiction construct. In any case, certain foods (fatty, sugary and salty) have shown to have an addictive potential, thus implying the possibility of preventing and treating obesity.

## Abbreviations

DA: dopamine
NAc: nucleus accumbens
BMI: body mass index
MC: melanocortins
YFAS: Yale Food Addiction Scale
PEMS: Palatable Motives Eating Scale
